# Micro-computed tomography of pulmonary fibrosis in mice induced by adenoviral gene transfer of biologically active transforming growth factor-β1

**DOI:** 10.1186/1465-9921-11-181

**Published:** 2010-12-22

**Authors:** Thomas Rodt, Christian von Falck, Sabine Dettmer, Roman Halter, Regina Maus, Kjetil Ask, Martin Kolb, Jack Gauldie, Florian Länger, Ludwig Hoy, Tobias Welte, Michael Galanski, Ulrich A Maus, Jürgen Borlak

**Affiliations:** 1 Department of Radiology, Hannover Medical School, Hannover, Germany; 2 Department of Molecular Medicine and Medical Biotechnology, Fraunhofer Institute for Toxicology and Experimental Medicine, Hannover Germany; 3 Department of Experimental Pneumology, Hannover Medical School, Hannover, Germany; 4 Department of Medicine, Pathology and Molecular Medicine, McMaster University, Hamilton, Canada; 5 Institute of Pathology, Hannover Medical School, Hannover, Germany; 6 Institute of Biometry, Hannover Medical School, Hannover, Germany; 7 Clinic for Pneumology, Hannover Medical School, Hannover, Germany

## Abstract

**Background:**

Micro-computed tomography (micro-CT) is a novel tool for monitoring acute and chronic disease states in small laboratory animals. Its value for assessing progressive lung fibrosis in mice has not been reported so far. Here we examined the importance of in vivo micro-CT as non-invasive tool to assess progression of pulmonary fibrosis in mice over time.

**Methods:**

Pulmonary fibrosis was induced in mice by intratracheal delivery of an adenoviral gene vector encoding biologically active TGF-ß1 (AdTGF-ß1). Respiratory gated and ungated micro-CT scans were performed at 1, 2, 3, and 4 weeks post pulmonary adenoviral gene or control vector delivery, and were then correlated with respective histopathology-based Ashcroft scoring of pulmonary fibrosis in mice. Visual assessment of image quality and consolidation was performed by 3 observers and a semi-automated quantification algorithm was applied to quantify aerated pulmonary volume as an inverse surrogate marker for pulmonary fibrosis.

**Results:**

We found a significant correlation between classical Ashcroft scoring and micro-CT assessment using both visual assessment and the semi-automated quantification algorithm. Pulmonary fibrosis could be clearly detected in micro-CT, image quality values were higher for respiratory gated exams, although differences were not significant. For assessment of fibrosis no significant difference between respiratory gated and ungated exams was observed.

**Conclusions:**

Together, we show that micro-CT is a powerful tool to assess pulmonary fibrosis in mice, using both visual assessment and semi-automated quantification algorithms. These data may be important in view of pre-clinical pharmacologic interventions for the treatment of lung fibrosis in small laboratory animals.

## Background

Pulmonary fibrosis is a severe, chronic lung disease, which is associated with high mortality rates, afflicting more than five million patients worldwide [1]. Its most severe form, idiopathic pulmonary fibrosis (IPF) is typically associated with an average survival of 2-5 years from time of diagnosis [2,3]. Depending on the underlying cause, progression of pulmonary fibrosis is associated with alveolar epithelial cell injury and hyperplasia, inflammatory leukocyte accumulations within the bronchoalveolar space and lung parenchymal tissue, as well as fibroblast hyperplasia and exuberant deposition of extracellular matrix (ECM) components such as collagen and fibronectin in the lungs, eventually leading to formation of honey comb cysts and scar tissue [2,4]. Specifically in IPF, immunosuppressive and anti-inflammatory therapies have proved to be ineffective in preventing the fatal consequences of lung tissue remodelling characterizing pulmonary fibrosis.

Over the past few years, it has become evident that animal model systems to study the pathogenesis of lung fibrosis in rodents need to be more carefully evaluated with respect to their applicability to human lung fibrosis [4]. As an example, application of the anti-neoplastic drug bleomycin into the lungs of mice or rats is the most frequently employed model system to study the pathogenesis of this devastating lung disease in small laboratory animals. However, in contrast to human lung fibrosis frequently developing over years and decades, bleomycin treatment of mice is characterized by early alveolar epithelial cell injury and acute lung inflammation followed by a transient lung fibrotic response, thereby not adequately reflecting human disease progression [4]. Another previously described model system to initiate fibrosis in small laboratory animals makes use of somatic cell gene transfer of biologically active transforming growth factor-β1, which, due to its unique fibrogenic characteristics, has been shown to trigger slowly progressing lung fibrosis in rats, which is irreversible and lacks acute inflammatory episodes [5].

Diagnostic tools to assess the degree of lung fibrosis in humans currently include lung CT scans, lung function tests and histological examinations of lung tissue biopsies. In mice, where complete lungs can be removed, particularly histological and biochemical approaches are employed to assess the degree of fibrosis [6,7]. In recent years, small animal imaging has emerged as an innovative and powerful, non-invasive tool to perform follow up of both acute and chronic disease processes in laboratory animals over time [8-11]. Various non-invasive techniques such as micro-CT, micro-positron emission tomography (micro-PET), and magnetic resonance imaging (MRI) are innovative tools that may be useful for evaluation of novel anti-fibrogenic therapeutic strategies in mouse models *in vivo*. However, systematic micro-computed tomography-based analysis of developing lung fibrosis induced by gene transfer of biologically active transforming growth factor-β1 as compared to classical histopathological approaches to monitor fibrotic disease progression in mice using both visual assessment and semi-automated quantification algorithms has not been reported so far.

Therefore, in the current study, pulmonary fibrosis was induced in mice by gene transfer of biologically active transforming growth factor-β1, and fibrotic disease progression was evaluated by micro-computed tomography and then correlated to histological scoring of lung fibrosis as pioneered by Ashcroft [12].

## Methods

### Adenoviral vectors

Two adenoviral vectors were employed, which were constructed as outlined in detail previously [5]. AdTGF-β1^223-225 ^contains the cDNA of the coding region of full-length porcine TGF-β1, while containing a mutation of cysteine to serine at positions 223 and 225, rendering expressed TGF-β1 biologically active [5]. Empty viral vector AdDL70-3 constructed as previously described was used as control [13].

### Induction of pulmonary fibrosis

**Figure 1:**
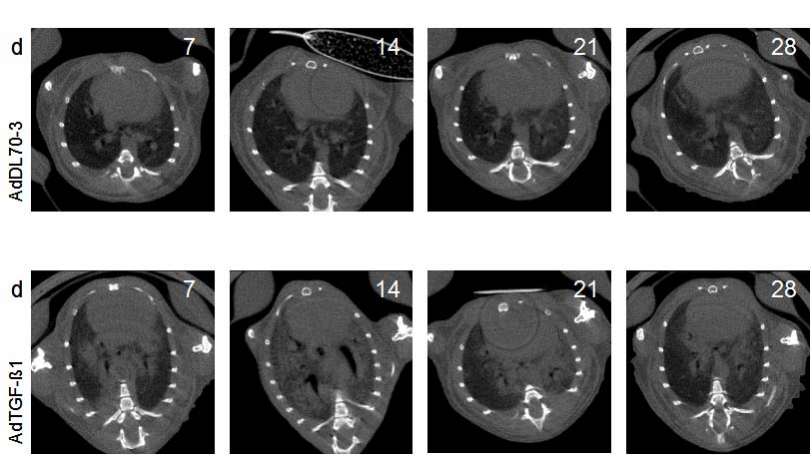
**Respiratory ungated in vivo micro-CT at 1, 2, 3 and 4 weeks after intratracheal instillation of control vector or AdTGFβ1**. (A) Lung micro-CT of mice treated with the control vector AdDL70-3. (B) Lung micro-CT of mice treated with AdTGF-β1. Only minor changes are seen in the control group, the consolidation in the fibrosis group is illustrated with pronounced micro-CT consolidation at 2 and 3 weeks. Representative images are shown.

Groups of female C57BL/6 mice (30 mice, age 8-12 weeks, weight 18-20 g) were either infected with AdTGF-β1^223-225 ^(herewith referred to as AdTGF-β1, 18 mice) or control vector AdDL70-3 (12 mice) via intratracheal instillation, as described recently in detail [6,14,15]. Briefly, anaesthetized mice were orotracheally intubated with an Abbocath (Abbott, Wiesbaden, Germany), followed by careful intratracheal instillation of adenoviral vectors (~1 × 10^8 ^plaque-forming units [PFU] per mouse) suspended in a total volume of 50 μl of phosphate-buffered saline (PBS). At 1, 2, 3, and 4 weeks after adenoviral transfer, mice (AdTGF-β1 for week 1-4: n = 3, n = 6, n = 6, n = 3; AdDL70-3 for week 1-4: n = 3 for each time point) were subjected to micro-computed tomography (Figure 1), followed by immediate sacrifice and isolation of lungs for histopathological evaluation of the degree of pulmonary fibrosis, using the scoring system described by Ashcroft et al. [12].

### Lung histology and assessment of pulmonary fibrosis

Complete lungs were inflated *in situ *with PBS-buffered (4.5%, pH 7.0) formalin (Roth, Darmstadt, Germany), and were then carefully removed and immersed in PBS-buffered formalin. Lung tissue samples were paraffin-embedded, and sections of 5 μm were prepared and stained with haematoxylin/eosin (H/E) and Masson's or Elastica-van-Gieson staining. The degree of lung fibrosis was determined according to the method described by Ashcroft and colleagues [12], employing a numerical scaling system in lung samples ranging from 0 (normal lung) to 8 (total fibrous obliteration of the field). The mean degree of lung fibrosis according to the definitions by Ashcroft et al. [12] was calculated from individual scores of ~15 microscopic fields analyzed per mouse lung.

### Micro-CT scans of mice

A GE Explore Locus cone-beam micro-CT (GE Healthcare, Chalfont St Giles, GB) was used for data-acquisition in prone position under isoflurane inhalation anaesthesia (tube voltage 80 kV, tube current 450 μA, 0.094 mm effective pixel size) with and without respiratory gating (i.e. synchronization of acquisition of micro-CT projections with a timepoint in the respiratory cycle of the individual mouse). Scanning took approximately 10 and 20 min without and with respiratory gating, respectively. Respiratory monitoring was performed using a pressure transducer pad under the animal's chest. Images were reconstructed and assessed at a constant window width/window level (5000/2000).

### Assessment of fibrosis by observer rating scale

Three radiologists blinded to the experimental groups and respiratory gating strategies, independently assessed image qualities of all individual entire micro CT scans, using a rating scale ranging from 1 (very good) to 5 (insufficient) and the amount of lung consolidations using a rating scale ranging from 1 (no consolidation) to 5 (very pronounced consolidation).

### Post-processing

Due to the diffuse nature of fibrotic consolidations, direct quantification of the pathology of lung fibrosis by measurement of focal fibrotic lesions was not feasible. Therefore, quantification of aerated lung areas known to be compromized by developing lung fibrosis was used as a surrogate marker for progressive lung fibrosis. A region-growing segmentation algorithm was applied on each individual entire micro CT scan using the MevisLab software package (Mevis Research, Bremen, Germany) [16]. The algorithm uses the seed points as starting points. From these points the segmentation volume "grows", with the structures included in the volume depending on the mathematical parameters. The segmentation algorithm was originally developed for quantification of diffuse lung tumors in transgenic mice, and was slightly modified for its use in the current study. Segmentation was carried out using 20-40 seed points, the segmentation threshold tolerance of the region growing algorithm was set to 2%.

### Statistical analysis

All statistical computations have been performed using the statistical program SPSS 17 (SPSS Inc., Chicago, Ill., USA) The significance level of all tests has been set to 0.05 (5%). Because most of the observed variables were ordinal scaled we used non-parametric tests for statistical analysis. Significance of differences in image quality values were tested for all three observers in respiratory gated and respiratory ungated exams using a Wilcoxon signed ranks test. Furthermore, significance of differences in consolidation assessment values for respiratory gated and respiratory ungated exams was tested using a Wilcoxon signed ranks test for all three observers. Differences between the assessment of the 3 observers were evaluated comparing the 95% confidence interval for the mean for assessment values of the image quality for respiratory gated/ungated exams and the assessment values of the consolidation assessment for respiratory gated/ungated exams.

Correlation of the Ashcroft histology scores (assessing the degree of fibrosis) and the lung consolidation values (obtained by assessment of the images by three observers) was calculated for respiratory ungated/respiratory gated exams and all three observers using Spearman's rank correlation coefficient for ordinary scaled variables.

Correlation of the Ashcroft histology scores and the lung segmentation values (obtained by the semiautomated segmentation routine described above) was calculated for respiratory ungated and respiratory gated exams using a Pearson correlation for continuous variables. To assess differences between the 4 time points for histology scores, visual consolidation assessment and segmentation of aerated lung areas 95% confidence intervals were plotted against the 4 time points and differences were tested for significance.

## Results

### In vivo micro-CT imaging of mouse lungs after intrapulmonary delivery of control vector or AdTGFβ1

As shown in Figure 1 micro-CT imaging of mouse lungs subjected to treatment with control vector or application of AdTGFβ1 for up to 4 weeks revealed that no major consolidations were detectable in control vector treated mice, whereas AdTGFβ1 treatment of mice provoked strong consolidations easily detectable particularly on weeks 2 and 3 post-treatment (Figure 1).

### Assessment of image quality and consolidations in respiratory gated as compared to ungated exams of fibrosing lungs

We next questioned whether detection of consolidations in fibrosing mouse lungs would differ between observers. However, initial comparative image quality assessment between three different observers revealed only slightly increased mean image quality values in respiratory gated (MV_SD _2.70, MV_CVF _2.33, MV_TR _2.27) as compared to ungated exams of fibrosing lungs (MV_SD _2.33, MV_CVF _2.10, MV_TR _1.97). Moreover, all three observers determined similar consolidation assessment values for respiratory gated (MV_SD _2.13, MV_CVF _2.40, MV_TR _2.30) as compared to ungated exams (MV_SD _2.13, MV_CVF _2.43, MV_TR _2.40).

Regarding the assessment variability between the 3 observers, we found that the lower and upper bound of the 95% confidence interval for the mean for the 3 observers showed no relevant differences except for the image quality assessment in gated exams. Here, lower and upper bound of the 95% confidence interval for the mean were distinctly higher for observer SD. The Cohen's kappa statistic also showed significant agreement between nearly all observer scores, but no significant agreement was found for the image quality evaluation scores by observers SD and TR for gated (p = 0.051) and ungated exams (p = 0.699).Overall, these data illustrate that visual assessment of micro-CT based lung fibrosis in mice was independent of the observer.

### Correlation of histological grading of lung fibrosis with micro-CT based fibrosis grading

**Figure 2:**
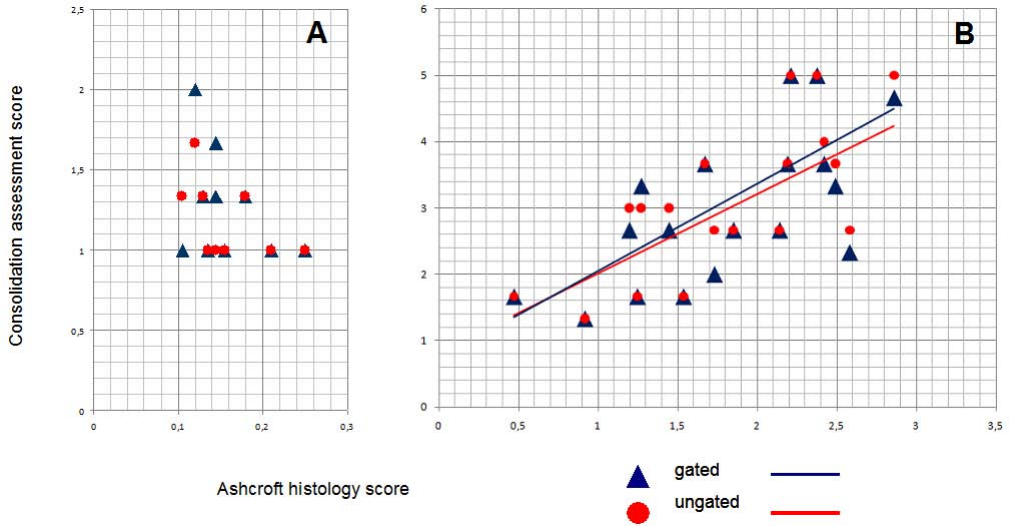
**Correlation of visual consolidation assessment and histological assessment by Ashcroft scoring in animals after treatment with control vector AdDL70-3 (A, showing very low Ashcroft histology scores (i.e. no fibrosis), and low visual consolidation assessment scores) or AdTGF-β1 (B)**. Mean consolidation assessment values of the 3 observers are plotted against Ashcroft scores of each animal in respiratory gated and respiratory ungated exams, as indicated. Linear interpolation is given for respiratory gated and ungated exams, respectively. Note the different scaling.

**Figure 3:**
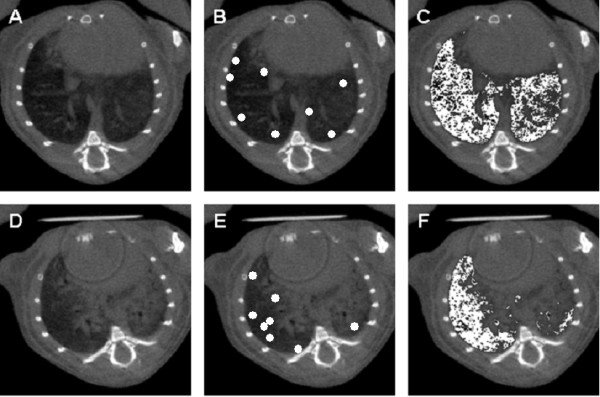
**Semiautomated region growing segmentation for assessment of pulmonary consolidation 3 weeks after instillation of control vector AdDL70-3 (A-C) or AdTGF-β1 (D-E)**. Aerated lung volume is used as an inverse surrogate marker for pulmonary fibrosis. (A, D) Axial micro-CT. (B, E) Placement of seed points for the region growing segmentation in the aerated lung. Seed points are enlarged for better detection. (C, F) Segmentation result shows aerated lung volume in white.

**Figure 4:**
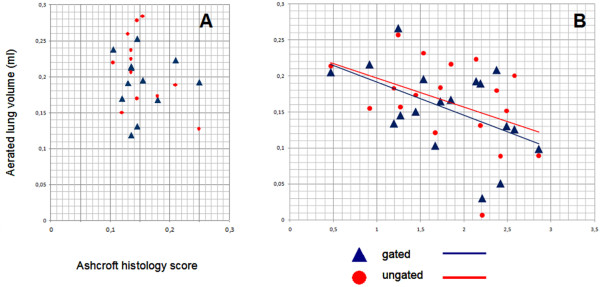
**Correlation of aerated lung volume determined by region growing segmentation with histological assessment of lung fibrosis by Ashcroft scoring in mice treated with control vector (A, showing very low Ashcroft histology scores (i.e. no fibrosis) and normal aerated lung volume) or AdTGF-β1 (B)**. Aerated lung volumes are plotted against Ashcroft scores for each animal in respiratory gated and respiratory ungated exams, as indicated. Linear interpolation is given for respiratory gated and ungated exams, respectively. Note the different scaling.

We next compared classical Ashcroft scoring with micro-CT based evaluation of TGFβ-1 induced lung fibrosis in mice. Ashcroft histology scores and lung consolidation values showed significant correlations for all observers and respiratory gated as well as respiratory ungated exams using Spearman's rank correlation coefficient (p < 0.001, Figure 2). Moreover, we also assessed progression of pulmonary fibrosis in mice by making use of a semi-automated region growing segmentation algorithm, where aerated lung volume was used as a surrogate marker for pulmonary fibrosis, which is illustrated in Figure 3. Ashcroft histology scores and lung segmentation values showed significant negative correlation using a Pearson correlation test for respiratory gated (p = 0.004) and respiratory ungated exams (p = 0.006) (Figure 4).

Due to the limited number of study objects within each group (AdTGF-β1 for weeks 1-4: n = 3, n = 6, n = 6, n = 3), we were not able to detect significant differences between different time points for visual consolidation assessment and segmentation of aerated lung areas. However, to better exemplify the time course of fibrosis in mice on days 7, 14, 21, and 28 post treatment, mean values and standard deviations for Ashcroft histology scores and visual assessment of all three observers for ungated exams are given (histology for control group: 0,15 ± 0,03; 0,14 ± 0,01; 0,17 ± 0,07; 0,15 ± 0,05/histology for fibrosis group: 1,79 ± 0,58; 2,13 ± 0,5; 1,67 ± 0,84; 1,64 ± 0,45/visual consolidation assessment scores for control group: 1,11 ± 0,19; 1,11 ± 0,19; 1,22 ± 0,38; 1,11 ± 0,19/visual consolidation assessment scores for fibrosis group: 3,56 ± 0,58; 4,06 ± 1,11; 2,50 ± 0,89; 2,00 ± 0,72).

## Discussion

In this study we evaluated the use of micro-CT for assessment of pulmonary fibrosis induced by adenoviral gene transfer of biologically active TGF-β1 in mice. We found micro-CT imaging to be a highly valuable tool to study pulmonary fibrosis progression in mice using both visual inspection of lung images and a semi-automated segementation algorithm that estimates aerated lung areas as a surrogate marker inversely correlating with developing lung fibrosis in mice.

Micro-CT allows a detailed assessment of lung morphology due to high density differences and well defined borders between air and lung tissue. However, one limitation of this method is that the activity of inflammatory processes can only be assessed by secondary characteristics by micro-CT, such as affection of adjacent strucutre or pleural effusion. Other imaging techniques such as magnetic resonance imaging (MRI), micro-positron emission tomography (PET) and optical imaging could provide functional data, with micro-PET beeing an especially promising candidate to robustly assess inflammatory activity by increase of glucose metabolism as detected by ^18^F-fluoro-deoxyglucose micro-PET [17-19], which might be particularly relevant in terms of monitoring acute exacerbations of pulmonary fibrosis in small laboratory animals.

Image quality is of equal importance in small animal imaging as in human imaging. However, data on imaging protocols are still not extensive. One major issue addressed in previous studies relates to respiratory gating. Various gating techniques, including prospective and retrospective as well as intrinsic and extrinsic techniques have revealed that mostly, gated imaging resulted in increased image quality [20,21]. In the current study, we found that mean values for image quality were better in gated exams for all observers, however differences were minor and not significant. Both gated and ungated exams resulted in a highly significant correlation with histology-based Ashcroft scores. Therefore, we conclude that gating should be applied whenever possible, but is not an absolutely critical issue when this setup is not available.

A further important aspect of micro CT imaging of normal or fibrosing mouse lungs is the time-point in the respiratory cycle when images are acquired. Due to the typical respiratory pattern in anesthesia, with a short breath followed by a long expiratory plateau, imaging is mostly performed in expiration. Under those experimental conditions, where intratracheal intubation and ventilation is performed, micro-CT imaging can also be performed in inspiration, as a different and consistent respiratory pattern can be created [22]. Without gating, imaging is effectively performed during expiration, due to the longer period of time as compared to inspiration that only represents a small fraction contributing to the average imaging time-point.

The semi-automated segmentation routine described in this study was found to assess consolidations in a quantitative way, as proven by the significant correlation of segmentation values with Ashcroft-based histological grading. To a minor degree, observer interaction is possible, as the observer has to place its own seed points for the region growing algorithm. As seed points have to be placed carefully within aerated lung areas, inadvertent positioning of seed points within non-aerated lung areas may increase the likelihood that consolidated aereas will be included in the segmentation volume and thus may cause assessment bias, which however, according to the presented data, appears to be very low.

In the current study, segmentation volumes were not correlated with total lung volume or body weight of individual mice. However, when considering that differences in expiratory total lung volumes between individual mice of the same age and strain are neglectable, it may be largely excluded that this aspect may have confounded the reported correlations. Future algorithms might take segmentation assessments of the entire lung into account, thereby allowing the correlation of measured consolidations with total lung volumes, or simply by employing a quotient taking the body weight into account.

Additionally, different techniques of fibrosis assessment could be applied. A promising approach is the measurement of CT densities expressed by Hounsfield Units and assessing fibrosis not only by consolidation but also by evaluation of density distribution [23]. Morphological pattern assessment as applied in human fibrosis assessment has also been described for imaging of lung fibrosis in small laboratory animals [24,25]. However, such approaches would be dependent on an image quality that is consistently comparable to human imaging, which is technically still difficult to achieve in *in vivo *imaging of small laboratory animals.

In another study we evaluated the applied radiation dose for the protocols used in this study applying phantom and cadaver thermoluminescence dosimetric measurements (own unpublished observations). The expected mean doses in an average C57BL/6 mouse for the respiratory gated and ungated protocols used in this study were 201 mGy and 194 mGy respectively. The expected mean value for an estimated time of 1 minute fluoroscopy to determine the scan field of view was 22 mGy. Day et al. reported that high radiation doses of 7 - 9 Gy did not result in significant lung fibrosis in mice after 90 days, just few microscopically small collagen-rich foci were detected subpleurally [26]. Therefore, it appears that radiation-induced fibrosis can be largely excluded even when serial examinations are performed.

Human lung fibrosis differs from the animal model reported here, as it is irreversible and shows typical morphological patterns in computed tomography representing definite fibrotic changes of the lung. The application of the methods reported for imaging in human lung fibrosis therefore is not intended. We believe that the currently applied technique will primarily be helpful to better assess e.g. novel therapeutic strategies for the treatment of pulmonary fibrosis in rodent model systems.

## Conclusion

In conclusion, the current study shows that respiratory gated/ungated micro-CT allows valid in vivo evaluation of the degree of pulmonary fibrosis in mice using both visual assessment and semi-automated quantification algorithms. Micro-CT thus offers a non-invasive imaging tool for evaluation of novel therapeutic strategies for the treatment of pulmonary fibrosis in mice.

## Competing interests

The authors declare that they have no competing interests.

## Authors' contributions

TR, CvF and UAM designed the study. TR, RM, RH and UAM carried out the preparation of the animals and micro-CT exams. TR, CvF and SD read and assessed the imaging studies. FL performed the histopathological workup. LH performed the statistical analysis. KA, MK, JG, TW, MG and JB participated in its design and coordination and helped to draft the manuscript. TR and UAM wrote the manuscript. All authors read and approved the final manuscript.
